# Analysis of 41 plant genomes supports a wave of successful genome duplications in association with the Cretaceous–Paleogene boundary

**DOI:** 10.1101/gr.168997.113

**Published:** 2014-08

**Authors:** Kevin Vanneste, Guy Baele, Steven Maere, Yves Van de Peer

**Affiliations:** 1 Department of Plant Systems Biology, VIB, Ghent B-9052, Belgium;; 2 Department of Plant Biotechnology and Bioinformatics, Ghent University, Ghent B-9052, Belgium;; 3 Department of Microbiology and Immunology, Rega Institute, KU Leuven, Leuven B-3000, Belgium;; 4 Department of Genetics, Genomics Research Institute, University of Pretoria, Pretoria 0002, South Africa

## Abstract

Ancient whole-genome duplications (WGDs), also referred to as paleopolyploidizations, have been reported in most evolutionary lineages. Their attributed role remains a major topic of discussion, ranging from an evolutionary dead end to a road toward evolutionary success, with evidence supporting both fates. Previously, based on dating WGDs in a limited number of plant species, we found a clustering of angiosperm paleopolyploidizations around the Cretaceous–Paleogene (K–Pg) extinction event about 66 million years ago. Here we revisit this finding, which has proven controversial, by combining genome sequence information for many more plant lineages and using more sophisticated analyses. We include 38 full genome sequences and three transcriptome assemblies in a Bayesian evolutionary analysis framework that incorporates uncorrelated relaxed clock methods and fossil uncertainty. In accordance with earlier findings, we demonstrate a strongly nonrandom pattern of genome duplications over time with many WGDs clustering around the K–Pg boundary. We interpret these results in the context of recent studies on invasive polyploid plant species, and suggest that polyploid establishment is promoted during times of environmental stress. We argue that considering the evolutionary potential of polyploids in light of the environmental and ecological conditions present around the time of polyploidization could mitigate the stark contrast in the proposed evolutionary fates of polyploids.

The omnipresence of whole-genome duplications (WGDs) in evolution is striking. Both angiosperm and vertebrate ancestors have undergone at least two separate WGDs, therefore all of their descendants are in fact ancient polyploids (paleopolyploids) (Putnam et al. 2008; Jiao et al. 2011). In the vertebrate lineage, a third WGD occurred in the ancestor of the successful teleost fish (Panopoulou and Poustka 2005). In the angiosperm lineage, subsequent and sometimes repeated WGDs have been reported in all major clades (Soltis et al. 2009; Van de Peer et al. 2009a). WGDs have also been documented in other kingdoms, such as, for instance, three WGDs in the ciliate *Paramecium tetraurelia* (Aury et al. 2006) and one WGD in the ancestor of the hemiascomycete *Saccharomyces cerevisiae* (Wolfe and Shields 1997). A systematic overview of WGD in invertebrates, amphibians, and reptiles is lacking, but several examples have been described, contradicting the classical notion that paleopolyploidies are absent in these lineages (Mable 2004; Song et al. 2012).

Although the prevalence of WGDs has been firmly established (Van de Peer et al. 2010), their attributed importance remains very controversial. Two long-standing opposite views regard polyploidy either as an evolutionary dead end (Stebbins 1950; Wagner 1970) or as a road toward evolutionary success (Levin 1983). Much research has been dedicated to this topic, especially in the plant lineage because of the high frequency of WGD occurrence in plants, and studies have typically found ample support for both scenarios. Recently formed polyploids frequently display increased meiotic and mitotic abnormalities through improper pairing of both subgenomes during cell division, resulting in genomic instability that has detrimental effects on plant fertility and fitness (Madlung et al. 2005). The study of mutant *Arabidopsis thaliana tam-1* plants that cannot enter meiosis II and therefore increase in ploidy in subsequent generations suggests that this genomic instability is polyploidy associated, as *tam-1* plants with higher ploidy levels exhibit more detrimental effects coupled with a strong drive to revert to lower ploidy levels via genomic reductions (Wang et al. 2010). Recently formed polyploid plants also need to cope with the minority cytotype disadvantage, a frequency-dependent reproductive disadvantage caused by ineffective matings of unreduced 2*n* gametes that cross with reduced *n* gametes from the diploid progenitor majority cytotype, which results in the formation of less fit and fertile triploid hybrids (Levin 1975). Consequently, even recently formed polyploids that are stable may be incapable of propagation because they simply cannot overcome the bottleneck of finding enough suitable mating partners to establish a viable population. Genomic and phenotypic instability, and the minority cytotype disadvantage, most likely contribute to the observation that polyploid plant species display lower speciation rates and higher extinction rates compared with diploids, and consequently, an overall lower net diversification rate (Mayrose et al. 2011).

In contrast, the fact that all extant angiosperms (Jiao et al. 2011) and vertebrates (Putnam et al. 2008) are paleopolyploids indicates that polyploidization is not always a dead end. Moreover, an estimated 15% and 31% of speciations in flowering plants and ferns, respectively, were accompanied by a ploidy increase (Wood et al. 2009). Most recent insights explaining the evolutionary success of polyploids have focused on their duplicated genome, which simultaneously provides thousands of novel genes for evolution to tinker with. Even though the large majority of these duplicated genes are lost through pseudogenization (Lynch and Conery 2000), the remaining fraction can lead to novel and/or expanded functionality through Ohno’s classical models of neofunctionalization (the duplicated copy acquires a new function), subfunctionalization (the division and/or elaboration of preduplication functionality over the two daughter copies), and gene conservation due to dosage effects (the increased production of a beneficial gene product), and combinations thereof (Ohno 1970; Hahn 2009; Maere and Van de Peer 2010). Interestingly, a fraction of WGD duplicates, including many regulatory and developmental genes, is most likely guarded against loss through dosage-balance constraints on the stoichiometry of duplicated pathways and/or macromolecular complexes (Maere et al. 2005; Freeling and Thomas 2006; Birchler and Veitia 2010). Resolution of dosage-balance constraints over time can thus provide polyploid species with an important toolbox that can be rewired to execute novel functionality (De Smet and Van de Peer 2012), and may allow them to cope with new ecological opportunities and/or challenges (Schranz et al. 2012; Fawcett et al. 2013). The ecological conditions that allow the initial establishment and long-term success of polyploids have been a major question in early polyploidy research for a long time, but progress in this regard has shifted somewhat to the background due to the explosion in research on their genomic composition (Soltis et al. 2010). Recently formed polyploids are traditionally considered to be good colonizers that have a broad ecological tolerance, which gives them an adaptive advantage as an invasive species (Thompson and Lumaret 1992; Otto and Whitton 2000). The latter can be attributed to their phenotypic instability, which can also be viewed as increased phenotypic variability and plasticity (te Beest et al. 2012). Such generalizations should, however, be treated with caution because of the paucity of large-scale systematic data on the subject and the many exceptions that can be found (Soltis et al. 2010).

In view of the contrasting WGD fates outlined above, it is perhaps not surprising that the precise nature of the link between WGD and evolutionary success remains heavily debated (Soltis et al. 2009; Abbasi 2010; Van de Peer et al. 2010). Previously, we performed absolute dating analyses on nine plant WGDs and proposed a link with the Cretaceous–Paleogene (K–Pg) extinction (Fawcett et al. 2009), which took place 66 million years ago (mya) according to the most recent estimates (Renne et al. 2013), suggesting that polyploidization somehow contributed to enhanced plant survival at that time (Fawcett and Van de Peer 2010). However, this study was limited in terms of taxonomic sampling due to the small number of plant genome sequences available at that time, and it relied on penalized likelihood inference methods that present inherent methodological challenges (Soltis and Burleigh 2009), such as, for instance, the assumption of an autocorrelated relaxed clock model that is most likely violated when taxon sampling is limited (Ho 2009). In the years since, the number of publicly available plant genomes has increased drastically, and the field of molecular dating has also progressed with the development of more powerful Bayesian methods of sequence divergence estimation that can incorporate advanced uncorrelated relaxed clock models and fossil age uncertainty (Drummond et al. 2006).

Here, we revisit the previously proposed clustering of plant paleopolyploidizations around the K–Pg boundary using the latest genome sequence data sets and phylogenetic dating methods available. We analyzed data from 41 plant species in total, including 38 full genome sequences and three transcriptome assemblies, to date 31 WGDs in various species that correspond to 20 independent plant WGDs. We used the BEAST software package, a state-of-the-art but computationally intensive Bayesian dating framework (Drummond et al. 2012). We tested whether these 20 plant WGDs follow a model where polyploid abundance simply increases randomly over time (Meyers and Levin 2006), or alternatively cluster statistically significantly in time in association with the K–Pg boundary (Fawcett et al. 2009), by comparing our WGD age estimates with a null model that assumes random WGD occurrence. We find a strongly nonrandom pattern with many WGDs clustering around the K–Pg boundary and we interpret our results in the light of new findings on recently formed plant polyploids that can help to explain this pattern. In particular, we argue that the environmental and ecological conditions during the time of polyploidization are of crucial importance.

## Results and Discussion

### Massive absolute dating of homeologs created through WGDs reveals the timing of plant paleopolyploidizations

**Figure 1.**
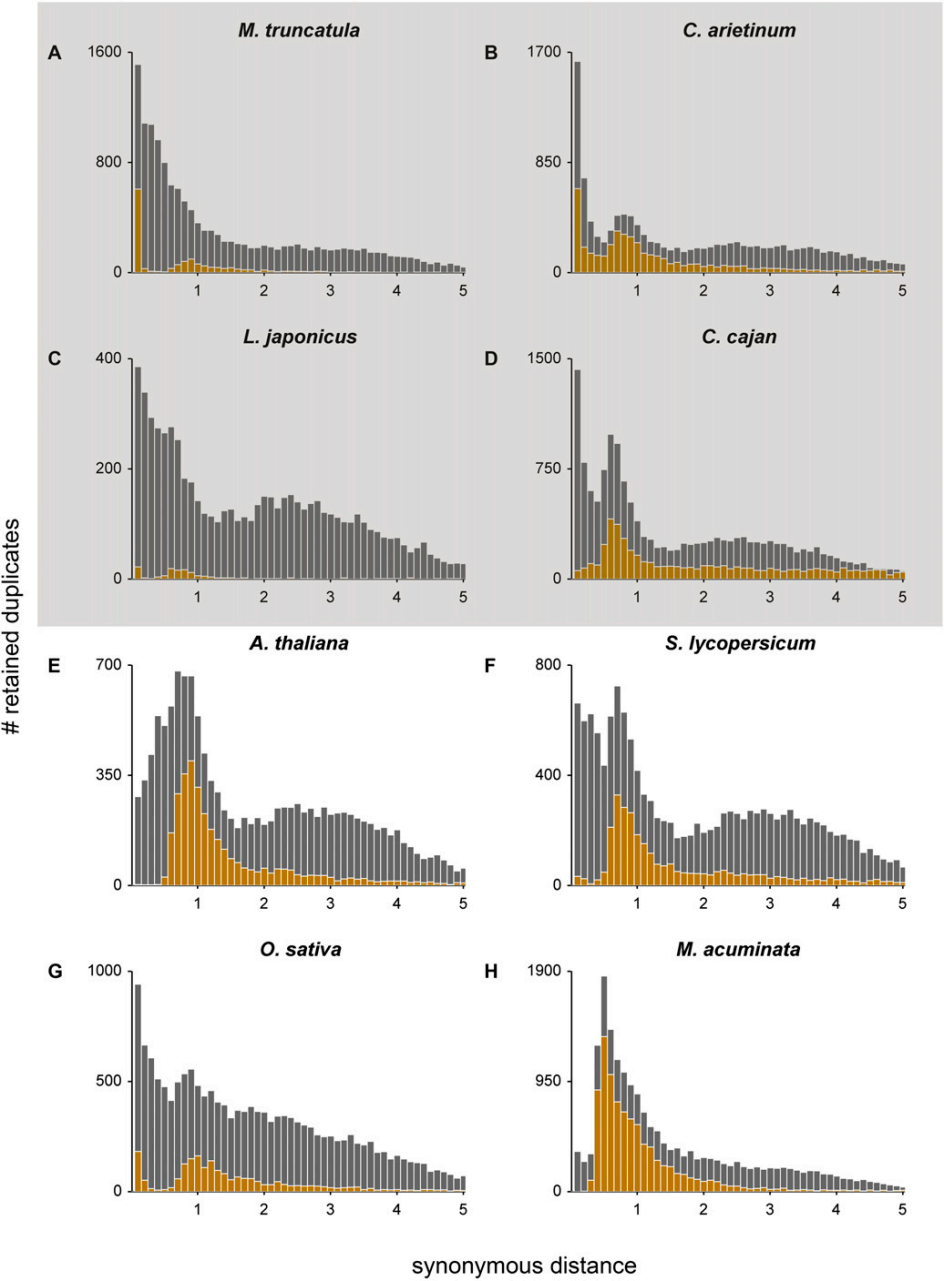
*K*_S_ age distributions for (*A*) *M. truncatula*, (*B*) *C. arietinum*, (*C*) *L. japonicus*, (*D*) *C. cajan*, (*E*) *A. thaliana*, (*F*) *S. lycopersicum*, (*G*) *O. sativa*, and (*H*) *M. acuminata*. The gray and gold bars represent the distribution of the paranome and duplicated anchors identified with i-ADHoRe, respectively. Anchors and peak-based duplicates used as homeologs for absolute dating were extracted from between the WGD peak boundaries (see Table 1). The gray box surrounding *A–D* indicates that these four species represent the same Faboideae-specific WGD.

**Figure 2.**
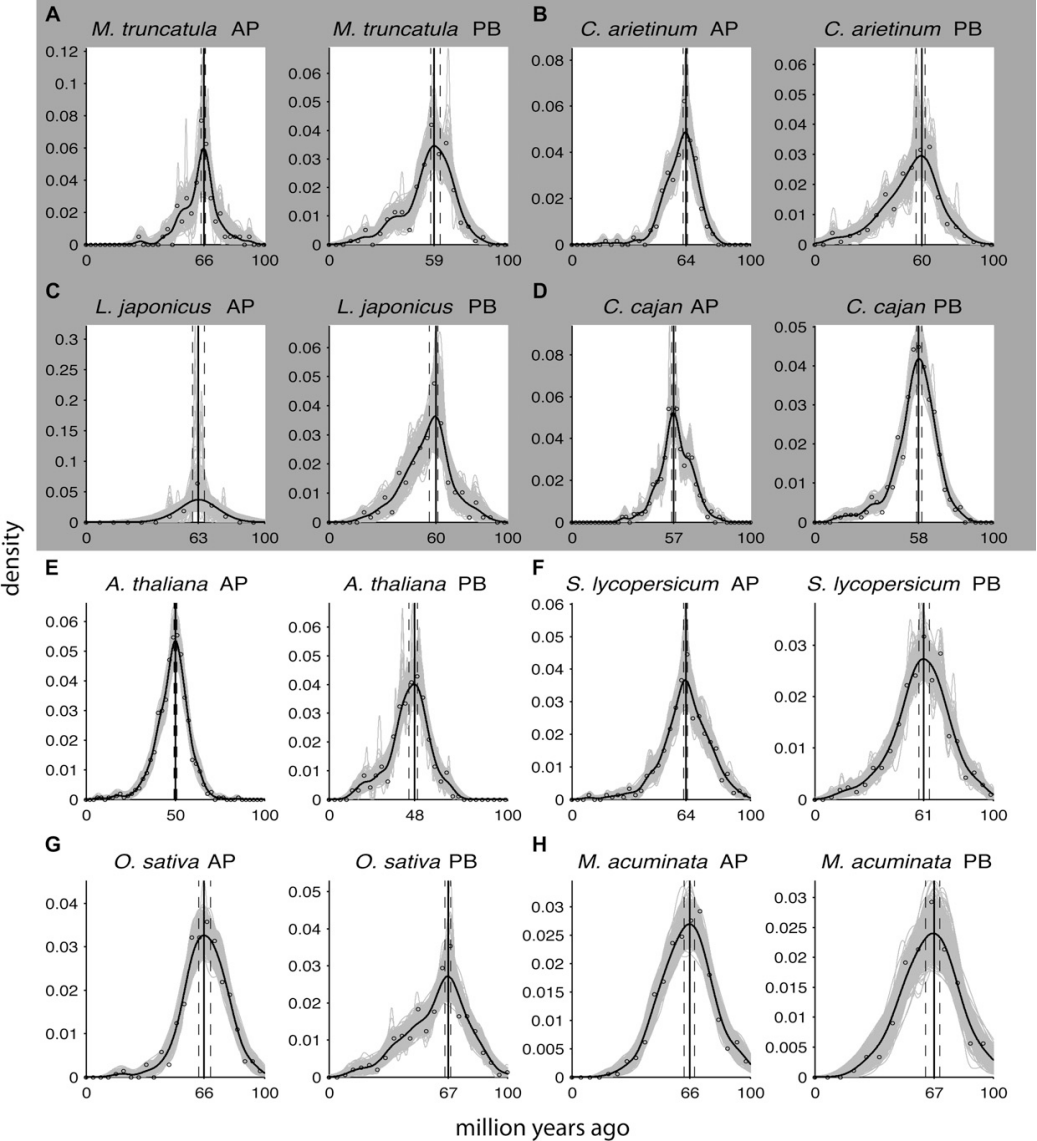
Absolute age distributions of the dated anchors (AP, *left*) and peak-based duplicates (PB, *right*) for (*A*) *M. truncatula*, (*B*) *C. arietinum*, (*C*) *L. japonicus*, (*D*) *C. cajan*, (*E*) *A. thaliana alpha* duplication, (*F*) *S. lycopersicum*, (*G*) *O. sativa*, and (*H*) *M. acuminata*. (Nonvertical black solid line) Kernel density estimate of the dated homeologs; (vertical black solid line) its peak used as the WGD age estimate. (Gray solid lines) Density estimates for the 1000 bootstrap replicates; (vertical black dashed lines) corresponding 90% confidence intervals on the WGD age estimate. The original raw distribution of dated homeologs is also indicated on the individual plots by open dots. See Table 1 for sample sizes and exact confidence interval boundaries. The gray box surrounding *A–D* indicates that these four species represent the same Faboideae-specific WGD.

**Figure 3.**
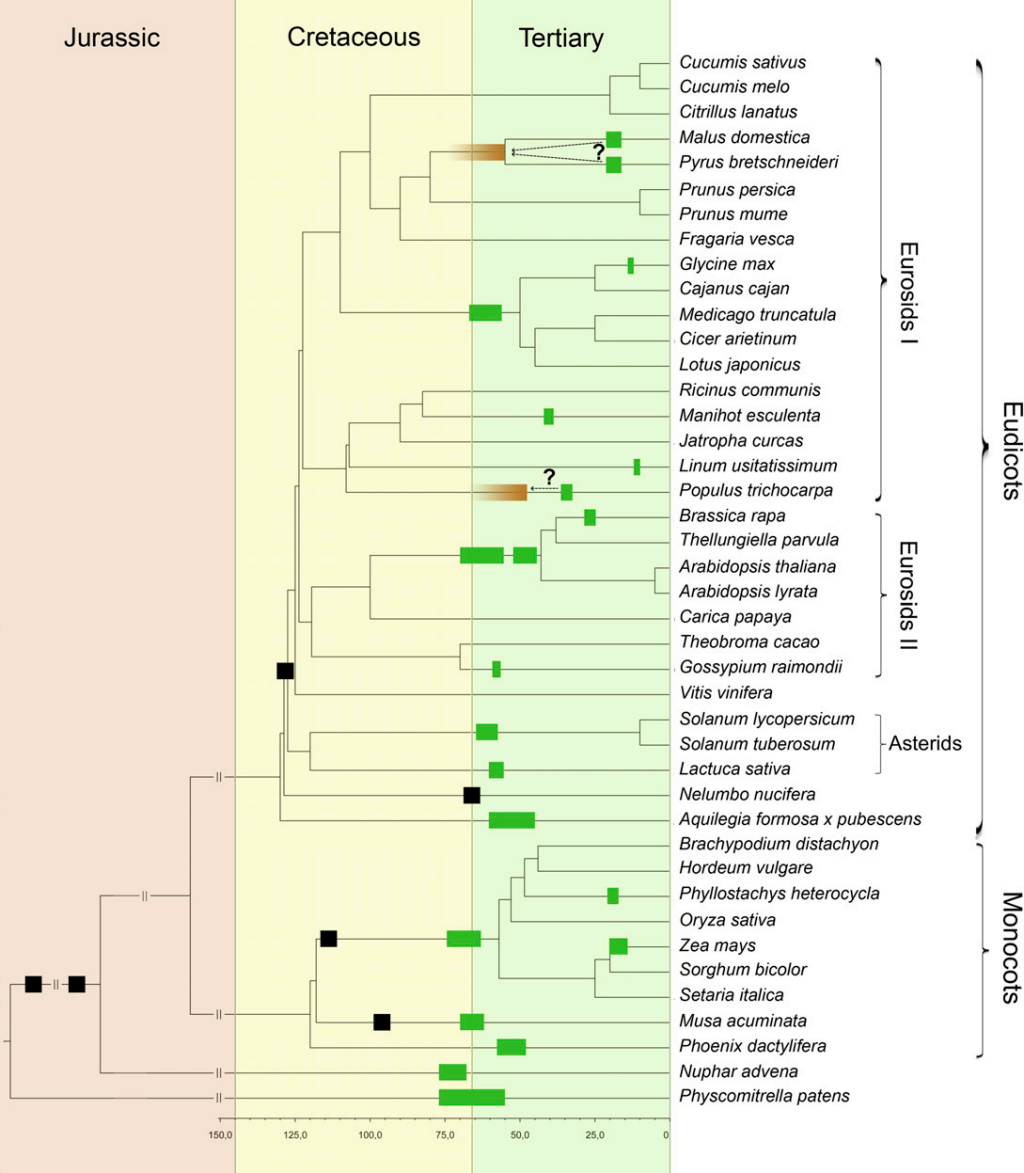
Phylogenetic tree of the green plants incorporating all species used in this study, with the exception of *N. nucifera*, which as a public annotation was not yet available upon completion. In total, sequence information from 38 full genome sequences and three transcriptome assemblies was used (see Supplemental Table S1). Bars indicate all known WGDs. Black bars indicate WGD age estimates from the literature and are not to scale (see Supplemental Information for justification and corresponding references). (Green bars) Estimates for WGDs dated in this study, with *right* and *left* boundaries corresponding to the youngest and oldest 90% confidence interval boundary found in the complete set of species-specific WGD age estimates that descend from each independent WGD (see Table 1). Some WGDs in woody species such as *G. raimondii* (Malvales), *P. trichocarpa*, and *M. esculenta* (Malpighiales), and the WGD shared by both *M. domestica* and *P. bretschneideri* (Rosales), are most likely underestimated through strong rate deceleration that is not fully corrected for (see Results and Discussion; Supplemental Information). The fading brown bars for the WGD in *P. trichocarpa*, and the WGD shared by *M. domestica* and *P. bretschneideri*, indicate corrected WGD age suggestions based on fossil evidence and/or other dating studies (see Results and Discussion). The green bar for *M. acuminata* most likely represents two separate WGDs in close succession (D’Hont et al. 2012). A possible WGD at the base of the monocots is not indicated because its exact phylogenetic placement remains unclear (Paterson et al. 2004). Branch lengths are truncated after 150 mya to improve clarity.

We focused on dating the most recent WGD in each plant species, because these can be most easily identified based on collinearity information (see Methods). One exception is *A. thaliana*, for which we were able to find a crude WGD age estimate for the older *beta* duplication, in addition to the more recent *alpha* duplication (Bowers et al. 2003), because of the high-quality genome sequence information available for this model species. Another special case is *Musa acuminata*, which most likely experienced two separate WGDs in very close succession that are problematic to differentiate between and that were therefore treated as a single event (D’Hont et al. 2012). We used two approaches to collect homeologs (genes created by WGD) for absolute dating. First, we used positional information to select anchor pairs, i.e., homeologs located on duplicated segments generated through WGD, with ages corresponding to the WGD signature peak in the *K*_S_ age distribution (Vanneste et al. 2013). Second, for species without positional information, or if fewer than 1000 orthogroups (see below) could be constructed based on anchors, we supplemented the anchor pairs with “peak-based” duplicates, which are non-anchor pairs that also map to the WGD signature peak in the *K*_S_ age distribution and therefore are assumed to consist mainly of homeologs (Maere et al. 2005). The selection of homeologs for different plant species that experienced a WGD in the last ∼100 million years is illustrated in Figure 1 for a few exemplary species, and in Supplemental Figure S1 for all other species. Next, all collected homeologs were combined with orthologs from other plant genomes to construct orthogroups (see Methods). The node joining the homeologous pair in each orthogroup phylogeny, representing the WGD of interest, was then dated using the uncorrelated lognormal (UCLD) relaxed clock model implemented in the BEAST package (Drummond et al. 2006, 2012) based on several primary fossil calibrations (see below). The resulting absolute age estimates for all homeologs collected from the same species were afterward grouped into one absolute age distribution, separated into anchors and peak-based duplicates where applicable. A consensus WGD age estimate was obtained for every species by taking the location of its peak in the absolute age distribution, as identified through kernel density estimation (KDE), while 90% confidence intervals (CIs) were obtained through a bootstrapping procedure (see Methods). Absolute age distributions for the species illustrated in Figure 1 are presented in Figure 2, and in Supplemental Figure S2 for all other species. All WGD age estimates, their 90% CIs, and the number of dated orthogroups they were based on, are listed in Table 1 per species, for both anchors and peak-based duplicates. A general overview of all dated WGDs mapped on the green plant phylogeny is also presented in Figure 3.

**Table 1.**
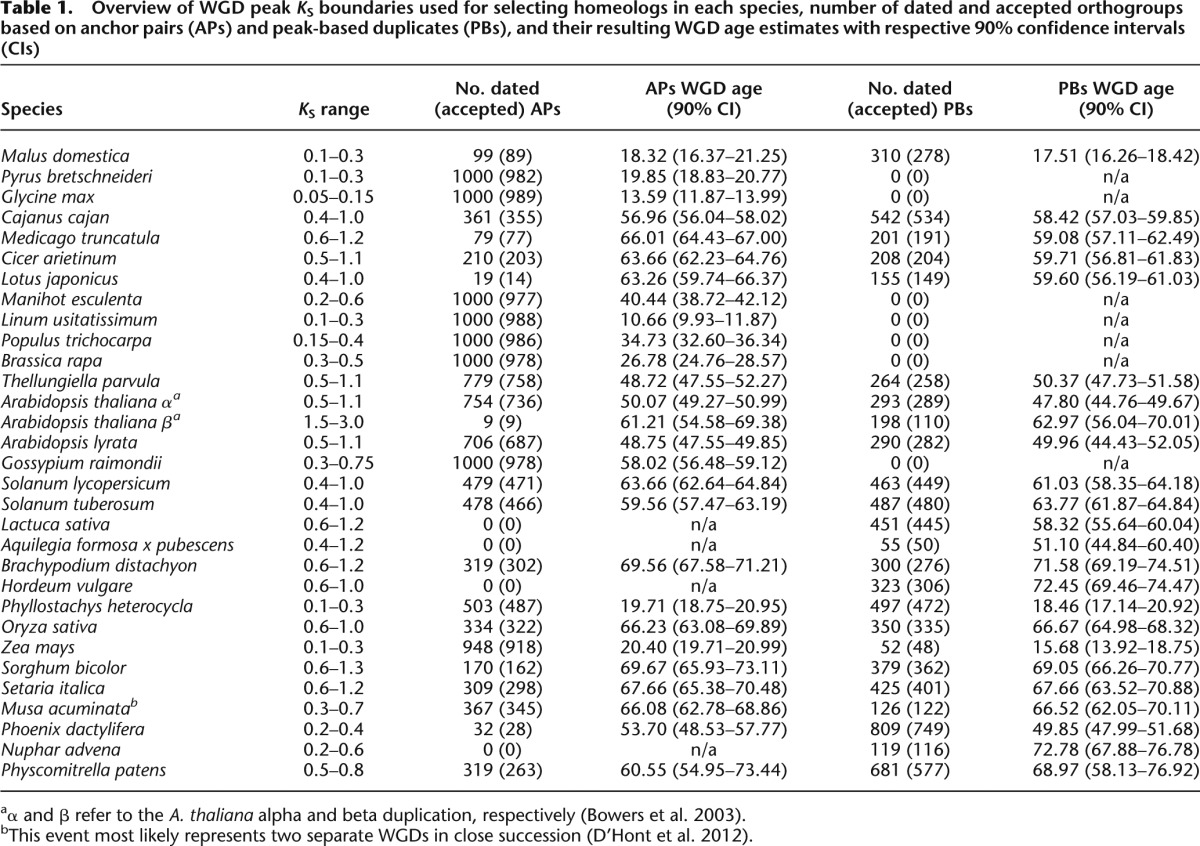
Overview of WGD peak *K*_S_ boundaries used for selecting homeologs in each species, number of dated and accepted orthogroups based on anchor pairs (APs) and peak-based duplicates (PBs), and their resulting WGD age estimates with respective 90% confidence intervals (CIs)

Figure 2 and Supplemental Figure S2 demonstrate that WGD age estimates obtained from absolute age distributions based on anchors and peak-based duplicates are in good agreement within the same species. However, the left flanks of peak-based absolute age distributions are denser compared with their right flanks, i.e., their distribution has a higher total probability of containing younger age estimates. This is most likely because a fraction of peak-based duplicates, namely those that do not derive from the WGD but from small-scale duplications in the timeframe covered by the WGD signature peak, follow an asymmetrical power-law distribution (Maere et al. 2005). As a result, the non-WGD pairs under the signature peak are slightly biased toward lower *K*_S_ values and younger ages. In contrast, anchor-based absolute age distributions exhibit a much more symmetrical shape. Nevertheless, KDE appears particularly well suited to correct for the different underlying shapes of anchor and peak-based absolute age distributions, and can accurately detect their peaks, which typically agree very well for both types of distributions within the same species. Their different shapes, however, prevent grouping both kinds of information into one absolute age distribution, despite the fact that anchors and peak-based duplicates theoretically describe the same species-specific WGD, since this would bias their resulting 90% CIs. Because anchor-based absolute age distributions are more symmetrical around their peak used for the WGD age estimate, and because they are based on actual duplicated segments, we consider them to be of higher quality, although peak-based duplicate WGD age estimates are clearly a good alternative for species where no or few anchors can be identified through lack of positional information.

In a few instances, we dated the same WGD in different descendant species. For instance, Figure 2 demonstrates the anchor-based absolute age distributions and resulting WGD age estimates for four species that diverged after the Faboideae-specific WGD (Doyle 2012): *Medicago truncatula* (66.01 mya), *Cicer arietinum* (63.66 mya), *Lotus japonicus* (63.26 mya), and *Cajanus cajan* (56.96 mya). Note that although *Glycine max* also shares this WGD, it underwent an additional more recent polyploidization, which we dated instead. The above four independent estimates converge on a WGD age of ∼63–66 mya, and also indicate that the *C. cajan* estimate most likely constitutes an underestimate, which might be due to either gene conversion or a strong genome-wide decelerated evolutionary rate that could not be completely corrected for (see below). Since all anchors from these four species describe the same event, an alternative strategy could have been to group them into one absolute age distribution to obtain a single WGD age estimate, which could, however, lead to misleading results. Since there are 361 dated anchors for *C. cajan* compared to 308 for all three other species combined (see Table 1), pooling them would introduce a systematic bias by pulling the whole absolute age distribution toward a younger WGD age estimate, and would also prevent us from inferring that the *C. cajan* WGD age most likely represents an underestimate. The same applies to peak-based duplicates that describe a shared WGD in other species. We expect that as new plant genomes become available, continued efforts in dating shared WGDs will help to pinpoint their exact age more precisely.

It should be noted that because allopolyploids result from the merger of two different species, in contrast to autopolyploids, their WGD age estimate could be slightly overestimated, because the latter reflects the time at which both contributing parental genomes started to diverge rather than the polyploidization itself (Doyle and Egan 2010). Distinguishing between auto- and allo-paleopolyploidizations is, however, notoriously difficult. Another caveat in estimating WGD ages is the influence of gene conversion, which may preserve WGD duplicates in an undiverged sequence state over extended time periods (Kellis et al. 2004; Sugino and Innan 2006), and would result in erroneously young WGD age estimates (Yang et al. 2012). Effects of such processes are very difficult to quantify for the large time scales considered in our data set, and their precise influence remains unknown.

### A substantial sequence compendium and state-of-the-art Bayesian evolutionary analysis framework increase confidence in our dating results

Our current study uses a substantially larger sequence compendium compared to our previous work (Fawcett et al. 2009), because only six full plant genomes (*A. thaliana*, *Populus trichocarpa*, *M. truncatula*, *Vitis vinifera*, *Oryza sativa*, and *Physcomitrella patens*) were available at that time, supplemented with a few transcriptome assemblies. We now incorporate sequence data from, in total, 38 full genome sequences and three transcriptome assemblies (see Supplemental Table S1). We originally included all transcriptome assemblies from the previous study, including *Eschscholzia californica* and *Acorus americanus* (Fawcett et al. 2009), but were unable to obtain unambiguous WGD age estimates for the latter with the methods used in this study (see Supplemental Information). In total, we could date 31 WGDs in various species that correspond to 20 independent WGDs in the plant lineage, previously compared with nine independent plant WGDs. Additionally, the typical orthogroup phylogeny size increased to a total of 14–15 sequences, previously compared to seven (Fawcett et al. 2009). The orthogroup size does not scale linearly with the total number of full plant genomes, because several species were grouped into species groups for which only one representative ortholog was included, in order to increase the total number of recovered orthogroups for dating (see Methods). The doubling of sequence information per orthogroup, in combination with a much broader coverage of the green plant phylogeny, are expected to improve the quality of the sequence signal that guides the molecular sequence divergence estimation (Yang and Rannala 2006; Rannala and Yang 2007; Mulcahy et al. 2012; Magallon et al. 2013).

Our previous work utilized the penalized likelihood inference method (Sanderson 2002), as implemented in the r8s package (Sanderson 2003), to date individual orthogroups (Fawcett et al. 2009), while the current study is based on a state-of-the-art Bayesian approach as implemented in the BEAST package, which incorporates several important methodological advances (Drummond et al. 2006, 2012). In particular, Markov chain Monte Carlo (MCMC) methods used in Bayesian sequence divergence estimation allow for much more parameter-rich and complex models of sequence evolution, and can also incorporate prior evidence and/or beliefs (Holder and Lewis 2003). This allows, for instance, for orthogroup branch lengths to be estimated together with other parameters during the MCMC, instead of having to estimate them a priori with other methods/software to avoid propagation of branch length errors (Thorne et al. 1998). However, of special importance is the more explicit modeling of both the underlying clock model and fossil calibration uncertainty (Yang and Yoder 2003).

Considering the underlying clock model, it is now generally accepted that molecular evolution does not follow a strict clock (Lanfear et al. 2010), particularly in the case for the evolutionary histories of the orthologs in the random orthogroups used here, which are expected to display a much larger degree of rate variation compared with the conserved housekeeping genes that are used in traditional molecular dating studies (Gabaldon and Koonin 2013). Since rates of evolution are linked to certain life-history traits such as generation time (Smith and Donoghue 2008), relaxed clock methods are preferable (Egan and Doyle 2010). Our previous work used an autocorrelated relaxed clock model (Fawcett et al. 2009), which assumes that adjacent branches share similar substitution rates because the latter are correlated with mutation rates that are affected by heritable life-history traits. These assumptions are, however, violated in case of sparse taxon sampling and when other forces such as selection are involved (Ho 2009; Smith et al. 2010). Moreover, even the very closely related *A. lyrata* and *A. thaliana* genomes exhibit a large degree of rate variation that can be attributed to other factors such as gene length, GC content, codon bias, and others (Yang and Gaut 2011). Similarly, large rate variation has been reported for homeologs stemming from the *alpha* WGD in *A. thaliana* (Zhang et al. 2002) and the WGD in *S. cerevisiae* (Scannell and Wolfe 2008). However, violation of the assumption of autocorrelation may lead to inconsistent estimates when using the penalized likelihood inference method (Mulcahy et al. 2012). Here, we use the UCLD relaxed clock model implemented in the BEAST package, which assumes an uncorrelated lognormal distribution of evolutionary rates (Drummond et al. 2006, 2012). The latter is a more realistic assumption in light of the above (Ho 2009; Smith et al. 2010), although a general consensus is still absent as at least one study found that autocorrelated clocks outperform uncorrelated clocks (Lepage et al. 2007), while another study found that both resulted in similar posterior age estimates (Magallon et al. 2013). Bayesian model testing methods that allow comparison of their performance exist (Baele et al. 2012, 2013), but applying them proved infeasible in terms of the required computational resources on the scale needed here (Baele and Lemey 2013).

Considering fossil calibration uncertainty, a substantial body of literature demonstrates that proper modeling of such uncertainty is of paramount importance because it allows for separation of the contribution of the evolutionary rate and total time to the overall observed divergence, which can heavily influence the posterior time estimates (Yang and Yoder 2003; Yang and Rannala 2006; Hug and Roger 2007; Inoue et al. 2010; Clarke et al. 2011; Mulcahy et al. 2012; Warnock et al. 2012; Magallon et al. 2013). Our previous work necessitated the use of mostly secondary point calibrations that were based on other molecular dating studies, because only limited opportunities for inserting primary calibrations based on direct fossil evidence were available (Fawcett et al. 2009). However, secondary calibrations carry the risk of propagating dating errors over different studies (Forest 2009), while point calibrations result in illusionary precision of the final age estimates (Ho and Phillips 2009). Our current study uses only primary fossil calibrations, modeled as flexible lognormal calibration priors that mimic the associated error in fossil calibration in an intuitive way (Forest 2009; Magallon et al. 2013). Orthogroup dating was always based on at least two calibrations. More calibrations allow for more rate corrections, and therefore help to guide molecular sequence divergence estimation (Benton and Donoghue 2007). At least one rate-correcting calibration was always present between the homeologous pair and root in all orthogroups, with the sole exception for dating the WGDs in *Nuphar advena* and *P. patens*, since their basal position necessitated a direct branch between the root and duplicate pair. Furthermore, the WGD age estimates presented in Table 1 are robust against differences in the utilized calibrations (see Supplemental Information).

### Some drastic rate shifts are not fully corrected for

Concerns have been raised that uncorrelated relaxed clocks still might not be able to correct completely for drastic rate shifts (Smith et al. 2010). To investigate the possibility of remaining rate-shift artifacts in our WGD age estimates, we performed pairwise relative rate tests (RRTs) between the different plant orders, using their respective full plant genomes that experienced a WGD where available, and found a mostly consistent pattern, particularly in the orders Malvales, Malpighiales, and Rosales which displayed a strong shift toward slower evolutionary rates (see Supplemental Information). This has been observed before as these three orders contain only woody species in our data set, while, in particular, woody status, large size, and long generation time have been associated with a strong decrease in evolutionary rate (Smith and Donoghue 2008; Korall et al. 2010; Lanfear 2011; Lanfear et al. 2013).

There is evidence that at least two WGDs for woody species in our data set most likely represent an underestimate. First, the *P. trichocarpa* (poplar tree) WGD constitutes a shared event of the genera *Populus* and *Salix*, both of which are members of the family Salicaceae within the order Malpighiales (Tuskan et al. 2006). The oldest known *Populus* fossils are leaves from the Middle Eocene Evacuation Creek at Green River Formation (Utah, USA) (Manchester et al. 1986, 2006), and are estimated to be at least 47.4 million years old (Boucher et al. 2003). Our estimate of 34.7 mya for the *P. trichocarpa* WGD (see Table 1) thus underestimates this boundary with at least 12.7 million years. Moreover, the latter is conservative because there exists an additional timespan between the shared WGD and divergence of *Populus* and *Salix* itself (Berlin et al. 2010). Second, the *Malus domestica* (apple tree) and *Pyrus bretschneideri* (pear tree) WGDs similarly constitute a shared event of the genera *Malus* and *Pyrus*, both of which are members of the family Rosaceae within the order Rosales (Wu et al. 2013). Fossil *Malus* and *Pyrus* leaves from the Eocene Orchards at Republic (Washington, USA) are, however, estimated to be at least 48.7 million years old (Wehr and Hopkins 1994). This age should be interpreted with due caution because fossil rosaceous leaves of closely related species are difficult to differentiate (DeVore and Pigg 2007), but it is supported by at least one molecular dating analysis focusing on these genera that estimated the divergence between *Malus* and *Pyrus* to be between ∼45 and 59 million years old (Lo and Donoghue 2012). Our two independent estimates for this shared WGD, 18.32 mya and 19.85 mya in *M. domestica* and *P. bretschneideri*, respectively, thus underestimate this boundary with at least ∼28 million years. The latter is again conservative because of the timespan between the shared WGD and actual divergence of both genera (Wu et al. 2013).

The above two examples demonstrate, perhaps not surprisingly, that strong rate shifts are still difficult to fully correct for by the uncorrelated relaxed clock model when taxon sampling is limited, but it remains difficult to quantify the effects thereof. We investigated this by specifically re-dating the *P. bretschneideri* WGD based on more complete taxon sampling and additional fossil calibrations that could be implemented for this particular species, and obtained a new WGD age estimate of 30.1 mya (see Supplemental Information). This constitutes an increase of more than 10 million years with respect to the original estimate, but still falls short by 18.6 million years of the previously described fossil minimum bound of 48.7 million years. This result suggests that breaking up long branches in orthogroup phylogenies through better taxon sampling, in combination with better rate-correcting fossil calibrations, will allow for correction of drastic rate shifts when more full plant genome sequences become available in the future. Note that the original WGD age estimate of *P. bretschneideri* is used in Table 1 and Figure 3 to allow for consistent comparison with the other WGD age estimates.

### Polyploid establishment was most likely enhanced at and/or after the K–Pg boundary

#### Plant paleopolyploidizations cluster statistically significantly in association with the K–Pg extinction

It has been proposed that a simple ratcheting process can explain the prevalence of polyploids. In essence, because polyploidization is an irreversible process, polyploid abundance is expected to increase over time (Meyers and Levin 2006). This ratcheting theory provides a null hypothesis to study paleopolyploid occurrence (Meyers and Levin 2006). In particular, it predicts that successful paleopolyploidizations are distributed randomly over time. We find, however, in line with previous results (Fawcett et al. 2009), that WGD age estimates exhibit a statistically significant clustering in time compared with a null model that assumes random WGD occurrence (*P* < 0.05, see Methods; Supplemental Fig. S3). Visual inspection of Figure 3 demonstrates that there is a large set of paleopolyploidizations that are situated relatively close to the K–Pg boundary. However, categorizing which specific WGDs can and cannot be considered as occurring in association with the K–Pg boundary is a difficult exercise. Because arbitrary cut-offs are susceptible to subjective bias, and are hence to be avoided, we chose to fit a mixture of Gaussian distributions to all WGD ages to judge the clustering timeframes statistically, and identified a pronounced component at 60.05 mya (see Methods; Supplemental Fig. S4). This suggests that a wave of WGDs occurred close to the K–Pg boundary, without making any a priori assumptions, but unfortunately also precludes making any post-hoc decisions about whether a particular WGD can be labeled as occurring at the K–Pg boundary or not.

This places many plant paleopolyploidizations at, but also especially after, the K–Pg extinction, which is the most recent of the five major mass extinctions of the Phanerozoic eon, during which an estimated ∼75% of all living species became extinct (Raup 1994). Several factors probably contributed to this large-scale extinction for an extended timespan, such as increased volcanism, greenhouse warming, and in particular the bolide impact near Chicxulub (Mexico) that marks the K–Pg boundary itself at 66.0 mya (Renne et al. 2013). Recent evidence indicates that this cataclysmic impact resulted in high levels of infrared radiation in the earth’s higher atmosphere, which led to worldwide firestorms that set whole ecosystems ablaze and created global dust clouds that blocked sunlight for an extended period of time (Robertson et al. 2013). This was most likely especially problematic for stationary plant communities, as evidenced by the extinction of about one-third to three-fifths of plant species (Wilf and Johnson 2004) and global deforestation (Vajda et al. 2001). The time interval for full plant community recovery was in the order of millions of years, and most early Paleogene localities are consequently characterized by an exceptionally low plant diversity (McElwain and Punyasena 2007). The overabundance of plant paleopolyploidizations at, and/or not long after, the K–Pg boundary indicates that polyploid establishment was enhanced during this period of mass extinction and/or recovery with respect to the simple ratcheting background model, which calls for potential explanations.

#### Enhanced polyploid establishment through increased adaptive potential under challenging conditions

Several adaptive advantages of possessing a polyploid genomic heritage for evolutionary innovations and/or species diversifications are being untangled (Schranz et al. 2012), but this long-term adaptive potential fails to explain why polyploids formed around the K–Pg boundary may have had a higher chance of establishment in the short term. Most explanations for the success of recently formed polyploids focus on their unstable genomic background which, despite most often leading to negative phenotypic effects through chromosomal abnormalities, also can infer the necessary plasticity to react quickly in a changing environmental context (Comai 2005). Typical short-term advantages include transgressive segregation and increased hybrid vigor, by which recently formed polyploids can display more extreme phenotypes than their diploid progenitors (Van de Peer et al. 2009b). This propensity for a broader ecological tolerance and increased invasive success in vacant and perturbed habitats was previously suggested as a potential explanation for the clustering of plant paleopolyploidizations at the K–Pg boundary (Fawcett et al. 2009).

There are some recent indications in favor of these adaptive hypotheses. Newly formed polyploids frequently display profound morphological and physiological differences (te Beest et al. 2012), and may have a higher capacity for phenotypic plasticity (Paun et al. 2011; Hahn et al. 2012) compared with their diploid progenitors. For instance, despite very low genetic diversity of the founder population, increased phenotypic plasticity most likely allowed polyploid *Ceratocapnos claviculata* species to recolonize northern European habitats after the last glacial maximum (Voss et al. 2012). Similarly, polyploid *Centaurea stoebe* species most likely displayed “pre-adaptation” for some traits that predisposed them for colonization success upon introduction in North America ∼120 yr ago (Henery et al. 2010). Polyploid *A. thaliana* plants have a broader salt tolerance, which may provide them with a fitness advantage that allows improved establishment in saline environments (Chao et al. 2013). Polyploids may even have a higher chance of being invasive, and diploids of being endangered, on a worldwide scale (Pandit et al. 2011). Such observations support the hypothesis that recently formed polyploids possess a propensity for a higher adaptive potential under challenging conditions, whereas the cost of increased phenotypic variability and genomic plasticity is most likely too high under “standard” conditions. This would explain why the signature of enhanced polyploid establishment upon drastic ecological upheaval, such as at the K–Pg boundary, is prominent enough to be picked up by our current, admittedly still limited, data and methods.

#### Enhanced polyploid establishment through mitigation of the minority cytotype disadvantage

A series of recent findings sketch an alternative explanation for enhanced polyploid establishment at the K–Pg boundary. The formation of unreduced 2*n* gametes is considered the main route toward polyploidization in plants (Harlan and De Wet 1975; Ramsey and Schemske 1998). Despite being traditionally viewed as too restrictive because of the low levels of unreduced gametes observed in natural plant populations, unreduced gamete production nevertheless appears adequate for cytotype coexistence in natural populations (Suda and Herben 2013). For instance, polyploid *Melampodium cinereum* populations originated recurrently since the last glacial maximum 12,000 yr ago in the Southwestern United States (Rebernig et al. 2010), illustrating that polyploids are indeed being formed continuously at an appreciable rate in stable environments. It is furthermore well established that environmental stress and/or fluctuations can even increase unreduced gamete formation in plants (Ramsey and Schemske 1998). The underlying molecular processes are being unraveled (De Storme and Geelen 2013b), and it appears that many of their associated components are thermosensitive (De Storme and Geelen 2013a). For instance, both heat stress in *Rosa* species and cold stress in *A. thaliana* led to increased unreduced gamete formation through alterations in spindle formation during meiosis II (Pecrix et al. 2011), and alterations in post-meiotic cell plate formation and cell wall establishment (De Storme et al. 2012), respectively. Similar observations exist in interspecific *Brassica* hybrids subject to cold stress (Mason et al. 2011), while most hybrids already exhibit increased levels of unreduced gamete formation (Ramsey and Schemske 1998). Recent evidence supports that environmental stress and/or fluctuations could also have increased unreduced gamete levels at previous large-scale extinctions, as demonstrated by the increased number of unreduced fossil pollen found in the now extinct conifer family Cheirolepidiaceae at the Triassic–Jurassic transition 201.3 mya (Kurschner et al. 2013). Abnormal gymnosperm pollen (Foster and Afonin 2005) and lycophyte spores (Visscher et al. 2004) have also been reported at the Permian–Triassic transition 252.3 mya (Shen et al. 2011). The former and latter boundary correspond to the second and third most recent mass extinctions in the Phanerozoic, respectively (Raup 1994).

These observations indicate that environmental stress and/or fluctuations can enhance plant polyploidization by promoting unreduced gamete formation. Alternatively, even in the absence of the latter, massive extinction of both diploid and polyploid cytotypes can decrease the overall plant population sizes markedly, which increases the role of stochastic drift in allowing it to overcome the minority cytotype disadvantage by random chance events (Mallet 2007). Both stress and extinction therefore have the potential to mitigate the minority cytotype disadvantage of polyploids by increasing their chances of finding suitable mating partners. Enhanced polyploid establishment under such conditions therefore does not necessarily require any direct adaptive advantage that promotes polyploid survival, but may rather be based on higher polyploid formation. This more neutral scenario is supported by modeling approaches that do not assume any a priori adaptive advantages of newly formed polyploids, but nevertheless find increased replacement of diploids by polyploids under a changing environment (Oswald and Nuismer 2011). Empirical observations also indicate that recently formed polyploids are much more abundant in stressful environments such as the Arctic (Brochmann et al. 2004), which might be due to both their adaptive potential and/or increased unreduced gamete formation (Mable 2004). Mitigating the minority cytotype disadvantage by increasing the polyploid minority cytotype frequency through increased unreduced gamete formation, and/or the influence of stochastic drift through overall background extinction of plant populations, does therefore constitute an alternative neutral explanation for the clustering of plant paleopolyploidizations at the K–Pg boundary that was not previously considered. Moreover, there exists a lag phase in the order of millions of years between the extremely stressful environmental conditions and the massive extinction associated with the K–Pg boundary itself, and plant population recovery afterward (Wilf and Johnson 2004; McElwain and Punyasena 2007), which effectively opens up an extended timespan during which the polyploid minority cytotype disadvantage was most likely alleviated. This would also explain why, apart from underestimated WGD ages through drastic rate shifts in some woody species (see before), plant paleopolyploidizations appear to cluster somewhat after the K–Pg boundary in a period characterized by slow recovery of plant population structure and size.

## Conclusion

In this study we dated 20 independent plant paleopolyploidizations. In line with previous results (Fawcett et al. 2009), we find that plant paleopolyploidizations in the last ∼100 million years are not distributed randomly over time but that many of them cluster in association with the K–Pg extinction boundary, which defies the hypothesis that successful polyploid establishment can be explained entirely by a simple ratcheting process. Given that our results are based on a substantial plant sequence information compendium with broad taxonomic coverage and a state-of-the-art Bayesian evolutionary analysis approach that incorporates uncorrelated relaxed clock models and fossil calibration uncertainty, this establishes the association of plant paleopolyploidizations with the K–Pg boundary as a legitimate hypothesis that warrants further investigation to either falsify or establish potential mechanistic explanations. In particular, we suggest that apart from traditional explanations for the success of recently formed polyploids that focus on their adaptive potential under sufficiently challenging conditions, more neutral mechanisms involving increased unreduced gamete formation and/or the influence of stochastic drift through background extinction merit further attention. We emphasize that our results do not support, nor do we claim, that WGD was either a prerequisite or guarantee for plant survival at the K–Pg boundary. Similarly, extinction and stress should not be viewed as absolute prerequisites or guarantees for successful polyploid establishment. We argue, however, that the establishment potential of polyploids should be viewed in light of the environmental and ecological challenges and opportunities at the time of polyploidization, in particular with stress and extinction being good candidate factors for promoting polyploid establishment. We believe that such a perspective will help to mitigate some of the conflicting hypotheses and observations on the proposed evolutionary fates of polyploids.

## Methods

### Data collection

In total, sequence information from 41 species was collected, including 38 full genome sequences and three transcriptome assemblies. A concise overview of utilized species and their data sources is provided in Supplemental Table S1. For annotated full genome sequences, protein-coding genes were used as provided by their respective annotations (all genes flagged as either suspected or known pseudogenes were removed). If alternative transcripts were available, only the one with the longest CDS was kept. For transcriptome assemblies, unigene sets were used as provided by their respective database. We used FrameDP (v1.0.3) (Gouzy et al. 2009) to extract the correct coding frame and putative coding sequence from the unigene sets, with Swiss-Prot (Bairoch et al. 2004) as a reference database for the underlying HMM model, and discarded genes shorter than 300 nt.

### Selection of homeologs

*K*_S_ age distributions for all species were constructed as described in Vanneste et al. (2013). For all species for which positional information was available, anchor pairs (i.e., duplicated gene pairs created by large-scale duplications that are positioned on duplicated segments) were extracted as follows. An all-against-all protein sequence similarity search was performed using BLASTP with an E-value cutoff of e^−10^. Paralogous gene pairs were retained if the two sequences were alignable over a length of more than 150 amino acids with an identity score of at least 30% (Li et al. 2001). Duplicated segments stemming from the most recent WGD were obtained by running i-ADHoRe (v3.0) (Fostier et al. 2011; Proost et al. 2012). i-ADHoRe parameters were set as follows: table_type = family, alignment_method = gg2, cluster_type = collinear, gap_size = 35, cluster_gap = 40, q_value = 0.75, prob_cutoff = 0.01, anchor_points = 3, multiple_hypothesis_correction = FDR, max_gaps_in_alignment = 40, and level_2_only = true. Peaks in the *K*_S_ age distribution supported by anchors were considered as valid WGD signatures. To ensure that all reported anchors were created by the WGD in question, only anchors on duplicated segments with median *K*_S_ values (calculated based on all anchors) between the WGD peak boundaries were accepted as homeologs. Paranome *K*_S_ distributions with anchors mapped on them are presented in Figure 1 for a few exemplary species, and in Supplemental Figure S1 for all other species. WGD peak *K*_S_ boundaries are presented in Table 1 for all species. For the Brassicaceae, we also tried to collect anchors for the older *beta* duplication (Bowers et al. 2003) by rerunning i-ADHoRe with level_2_only = false, but this approach only resulted in enough quality orthogroups (see next section) for *A. thaliana* because of its high-quality genome information. *M. acuminata* is a special case because its peak in the *K*_S_ age distribution most likely represents two WGDs in very short succession (D’Hont et al. 2012) so that anchors reported by i-ADHoRe most likely stem from two WGDs. We therefore treated the *M. acuminata* WGD peak as a single event (D’Hont et al. 2012).

For species where no or few anchors could be collected through lack of positional information due to a fragmented assembly or in case of transcriptome data, we used an alternative strategy to collect homeologs by selecting duplicate pairs from the WGD peak in the *K*_S_ age distribution. Although some of these duplicate pairs may not have been created by WGD, but rather by small-scale duplications in the same time frame, it can be safely assumed that the majority derives from the WGD (Maere et al. 2005; Vanneste et al. 2013). Because multiple paralogous pairs can descend from the same gene duplication due to subsequent duplications (Fawcett et al. 2009), we built amino acid-based phylogenies for all paralogous gene families in each species using PhyML (v3.0) (Guindon et al. 2010) with default parameters, which were rooted using a mid-point rooting approach (Hess and De Moraes Russo 2007). For duplication nodes with median *K*_S_ values (calculated based on all their terminals) between the WGD peak boundaries (see Table 1), a random pair of descendent genes was taken as the representative homeologous pair. This strategy was applied for all species where fewer than 1000 orthogroups (see next section) could be collected based on anchors, to increase the total number of homeologs used for obtaining a WGD age estimate.

### Orthogroup construction

For each collected homeologous pair, an orthogroup was constructed consisting of the homeologous pair and their orthologs in other plant species, since orthology relationships provide the most accurate representation of the followed evolutionary history (Fawcett et al. 2009; Altenhoff et al. 2012; Gabaldon and Koonin 2013). We used Inparanoid (v4.1) (Ostlund et al. 2010) with default parameter settings to detect orthologs. However, simply adding all identified orthologs from the other plant species to the homeologous pair was not feasible, because this would result in a plethora of possible tree topologies, for which applying the proper fossil calibrations and model specifications based on the BEAST XML syntax (see below) would be problematic. Additionally, this could also lead to systematic biases between different homeologous pairs from the same species caused by a different “tree context.” However, keeping the orthogroup topology fixed by requiring one ortholog to be present for every species listed in Supplemental Table S1 also proved problematic because this resulted in a drastic drop of the total number of recovered orthogroups, since most homeologs had to be discarded because orthologs could not be found in every other plant species. This is probably due to both species-specific ortholog loss and problems with orthology-detection performance, since the latter decreases together with genome annotation quality, especially over large evolutionary distances (Trachana et al. 2011), and many plant genomes have only been sequenced at relatively low coverage (Milinkovitch et al. 2010).

We therefore used a strategy where different species were put together in species groups, each consisting of two to four members. For each species group, the best ortholog (based on the average score reported by Inparanoid to both paralogs of the homeologous pair) was selected as the representative ortholog for that species group and added to the orthogroup. As a consequence, the orthogroup topology could be held constant, whereas for most homeologs at least one ortholog could be collected per species group so that the total number of recovered orthogroups for dating remained high and few homeologs had to be discarded. An extended description and justification for our use of a species grouping topology is provided in the Supplemental Information. Table 1 summarizes the total number of collected orthogroups, separated into anchors and peak-based duplicates per species, where applicable. Lastly, the homeologous pair was always fixed to cluster together in all orthogroups by not allowing any speciation after duplication scenarios. The latter would entail identifying the correct orthology relationships in sets of outparalogs, which is notoriously difficult (Koonin 2005; Brysting et al. 2007).

### Orthogroup dating

All sequences in each orthogroup were aligned using MUSCLE (v3.8.31) (Edgar 2004). Orthogroup alignments were cleaned up as described previously (Vandepoele et al. 2004), and only orthogroups with a cleaned alignment of more than 100 amino acids were retained for further analysis. We used BEAST (v1.7.4) (Drummond et al. 2012) to date the node joining the homeologous pair that represents the WGD of interest in each orthogroup. We set the underlying evolutionary model to be Le-Gascuel (LG), which is the most recent and large-scale amino acid replacement matrix available (Le and Gascuel 2008), with gamma-distributed rate heterogeneity across sites using four rate categories (Yang 1996). To this end, we have implemented the LG model into the BEAST source code, as this model was not yet publicly available. We used an uncorrelated relaxed clock model that assumes an underlying lognormal distribution (UCLD) on the evolutionary rates (Drummond et al. 2006), which is more likely to yield accurate estimates than the uncorrelated relaxed clock model that assumes an exponential distribution (UCED) on the evolutionary rates (Baele et al. 2013). A Yule pure birth process (Yule 1925) was specified for the underlying tree model because contemporaneous sequences are considered in all orthogroups. We utilized the following priors: a uniform prior between 0 and 100 for the Yule birth rate; an exponential prior with mean 0.5 on the rate heterogeneity parameter; an exponential prior with mean 1/3 on the standard deviation of the UCLD clock model; and a diffuse gamma prior with shape 0.001 and scale 1000 on the mean of the UCLD clock model. Priors on the fossil calibrations are detailed extensively in the Supplemental Information. A starting tree with branch lengths satisfying all of the fossil prior constraints was manually constructed and is also presented in the Supplemental Information. Operators on the tree model were disabled to keep the topology fixed so that only the branch lengths were optimized.

The MCMC analysis for each orthogroup was run for 10 million generations, while sampling every 1000 generations, resulting in a total size of 10,000 samples per orthogroup. The quality of the approximation of the posterior distribution improves as the number of generations, i.e., the amount of computational time devoted to the MCMC, increases (Lewis 2001; Sanderson et al. 2004). These methods are therefore computationally very intensive (Suchard and Rambaut 2009; Ayres et al. 2012), especially since we had to process a total of 22,252 individual evolutionary histories across all collected orthogroups. There exist faster implementations incorporating relaxed clock methods in a Bayesian context, but we still preferred the use of BEAST because it scores very high on benchmarks (Battistuzzi et al. 2011) and also has a very rich XML language syntax. We used a strategy where the separate orthogroups were run distributed over multiple CPU cores for independent evaluation (Moret et al. 2002). We also made use of the BEAGLE library, which speeds up the MCMC by taking over part of the core likelihood calculations (Ayres et al. 2012). Since visual inspection of each individual trace file for each orthogroup was impossible, we used LogAnalyser (part of the BEAST package) for automated evaluation of the orthogroups. A burn-in of 1000 samples was used and orthogroups were only accepted if the minimum effective sample size (ESS) for all statistics was at least 200. Table 1 summarizes the total number of accepted orthogroups, separated into anchors and peak-based duplicates per species, where applicable.

### Obtaining species-specific WGD age estimates

The age estimates for the node joining the homeologous pair in all accepted orthogroups were collected, and grouped into one or two absolute age distributions per species containing either age estimates based on anchors and/or peak-based duplicates, where applicable (see Table 1). A consensus WGD age estimate was obtained for each absolute age distribution by taking the mode of its kernel density estimate (KDE). The latter is much more flexible in comparison with traditional parametric distributions because it does not limit the shape of the estimated distribution to parameter-described forms, and therefore allows a much better exploration of the true underlying distribution and its trends (Botev et al. 2010). We utilized MATLAB (Release 2011a, The MathWorks Inc.) and the KDE toolbox (available at http://www.mathworks.com/matlabcentral/fileexchange/17204-kernel-density-estimation [retrieved March 21, 2013]), which allows automatic bandwidth selection (Botev et al. 2010). We used bootstrapping to obtain 90% confidence intervals (CIs) for all WGD age estimates (Hall and Kang 2001). For a data set of age estimates {*x*_*i*_; *i* = 1...*n*}, *n*-values are resampled with replacement to collect the bootstrap data set {*x*_i_*; *i* = 1...*n*} and KDE is performed on *x*_i_* to obtain the bootstrap density estimate 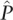
*. This is repeated 1000 times to collect a set of bootstrap density estimates {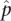 *; *j* = 1...1000}. The distribution of 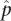
* around the original density estimate 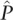 mimics the distribution of 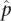 around the true density *p*, so that the modes for the 51st and 949th bootstrap density estimate (ranked in order of increasing value for their mode) give the lower and higher 90% CI boundary, respectively. Absolute age distributions are presented in Figure 2 for a few exemplary species, and in Supplemental Figure S2 for all other species. Exact values for species-specific WGD age estimates and their corresponding 90% CIs, separated into anchors and peak-based duplicates where applicable, are listed in Table 1.

### Clustering of WGD in time

Assessing whether there exists a statistically significant grouping of WGDs in time was based on the median distance between WGD age estimates as described in Fawcett et al. (2009). Briefly summarized, smaller median distances indicate a tighter clustering. The observed median distance between WGDs was compared with a null model that is based on random WGD occurrence by assuming a background distribution where the probability of WGD occurrence at a certain point in time is proportional to the total number of species present at that time (see Supplemental Fig. S3). One million random samples were pulled from this null model to assess the probability that the observed median distance is significantly lower than the distribution of median distances based on random WGD occurrence. We considered a timespan between 0 and 100 mya, as both the identification and timing of older paleopolyploidizations is still uncertain. All WGD age estimates listed in Table 1 were taken into account. Shared WGDs were only counted once by taking the average of WGD age estimates in all of their descendant species (see Fig. 3), always using anchor-based WGD age estimates and only peak-based WGD age estimates if the former were not available. The observed median distance was significantly lower than expected under the null model (*P*-value = 0.03, see Supplemental Fig. S3), indicating clustering of plant paleopolyploidizations in time. Moreover, this test is conservative because WGD age estimates in some woody species are most likely too young (see Results and Discussion).

This evaluation of clustering does not, however, identify the exact location of the clustering. Because any a priori criterion to associate WGDs with the K–Pg boundary would be based on arbitrary cut-offs, and is hence undesirable, we fitted a mixture of Gaussians (i.e., normal distributions) to the WGD age estimates (shared WGDs were only counted once as before) using the gmdistribution.fit function in MATLAB. According to the Akaike Information Criterion (AIC) (Akaike 1974), a mixture with two components had the best fit to the raw data (AIC = 174.90 compared with AIC = 180.33 and 177.96 for a mixture with one and three components, respectively). This mixture contained one very pronounced component at a location of 60.05 mya, corresponding to a clustering of WGDs close to the K–Pg boundary, while the second lesser component was located at 22.91 mya and most likely represents the background distribution (see Supplemental Fig. S4). Exclusion of the *M. acuminata* WGD in these analyses, because the latter most likely represents two WGDs in very close succession (D’Hont et al. 2012), did not significantly change these results (see Supplemental Figs. S3, S4).
